# Early-Career Scientists Shaping the World

**DOI:** 10.1128/mSystems.00196-19

**Published:** 2019-05-07

**Authors:** Ileana M. Cristea, Pieter C. Dorrestein, Jonathan A. Eisen, Jack A. Gilbert, Julie A. Huber, Janet K. Jansson, Rob Knight, Katherine S. Pollard, Jeroen Raes, Pamela A. Silver, Nicole S. Webster, Jian Xu

**Affiliations:** a Princeton University, Princeton, New Jersey, USA; b University of California, San Diego, La Jolla, California, USA; c University of California, Davis, Davis, California, USA; d Woods Hole Oceanographic Institution, Woods Hole, Massachusetts, USA; e Pacific Northwest National Laboratory, Richland, Washington, USA; f Gladstone Institutes, San Francisco, California, USA; g VIB-KU Leuven, Leuven, Belgium; h Harvard Medical School, Boston, Massachusetts, USA; i Australian Institute of Marine Science, Cape Ferguson, Queensland, Australia; j Qingdao Institute of BioEnergy and Bioprocess Technology, Chinese Academy of Sciences, Qingdao, China

## EDITORIAL

Last year we launched our first “Early-Career Special Issue” to highlight and share the work of some of the pioneers in systems microbiology science who are just stepping into their first independent research positions. The special issue—published in March/April 2018 (https://msystems.asm.org/content/3/2)—was, we believe, a great success. First, it provided a platform for these early-career researchers to highlight their work and their vision, in a format frequently reserved for people further along in their careers. Having such a large collection all in a single place allows one to get an excellent perspective on the future of microbial systems biology. Second, we believe the collection also serves as an important networking and communication resource. For example, conference organizers, companies, department chairs, and others looking for early-career scholars working across the breadth of microbial systems biology can use these articles. In addition, the collection provides an opportunity to see the bigger picture of the vision driving the work of key people in the field, which in turn could be a key resource for those looking for new collaborators, or possible mentors for graduate school or postdoctoral training.

A key part of the ethos of this special issue was to highlight and promote diversity of scientific ideas and approaches within microbial systems biology. We believe it is also important to note that diversity of the authors themselves (e.g., gender, type of institution, research background) was also an important aspect of organizing this special issue. We considered this particularly important because of implicit and explicit biases that affect authorship in STEM fields. When possible, it is thus important to counter such biases.

We realize the inaugural issue last year was neither comprehensive nor a perfect representation of all the diverse people, approaches, interdisciplinary topics, and viewpoints in the field of microbial systems biology. We are therefore pleased to provide the second special issue focused on early-career scientists in the field. We hope that you read, share, absorb, and reflect on the ideas, concepts, tool, and visions presented here. May they be a source of both inspiration and also collaboration with the young visionaries of our field.

